# Dupilumab for the treatment of atopic dermatitis in transplant patients: Two case reports and literature review

**DOI:** 10.1111/dth.15324

**Published:** 2022-01-30

**Authors:** Maddalena Napolitano, Mariateresa Nocerino, Vincenzo Picone, Fabrizio Martora, Gabriella Fabbrocini, Stefano Dastoli, Cataldo Patruno

**Affiliations:** ^1^ Department of Medicine and Health Sciences Vincenzo Tiberio University of Molise Campobasso Italy; ^2^ Section of Dermatology, Department of Clinical Medicine and Surgery University of Naples Federico II Naples Italy; ^3^ Department of Health Sciences University Magna Graecia of Catanzaro Catanzaro Italy

Dear Editor,

Dupilumab, a monoclonal antibody approved for the treatment of moderate‐to‐severe atopic dermatitis (AD), has a good safety profile.^1^ Its contraindications are very few, such as known hypersensitivity to dupilumab or any of the excipients.^2^ Currently, there are sporadic reports of dupilumab use in solid organ transplant recipients^3^, ^4^, ^5^, ^6^, ^7^ (Table 1). We report two transplant patients affected by AD and treated with dupilumab.

**TABLE 1:** Cases of atopic dermatitis patients treated with dupilumab after transplant

Age [ref]	Sex	Transplanted organ	Age at transplantation	Start dupilumab (years)	Outcome dupilumab	Associated immunosuppressive therapies
32 [3]	M	Renal	Not reported	32	Successfully treated	Not reported
12 [4]	F	Heart	2 years	12	Successfully treated	Tacrolimus and steroids
			7 years			
38 [5]	F	Liver	22 years	38	Successfully treated even if the patient developed persistent moderate conjunctivitis treated with glucocorticosteroid‐containing eye drops	Tacrolimus
18 [6]	M	Hearth	1 month	18	Successfully treated	Tacrolimus, prednisone and mycophenolate mofetil
29 [7]	F	Hearth	25 years	29	Successfully treated	Tacrolimus, prednisone and mycophenolate mofetil

The first, a 52‐year‐old male was referred to us for a 10‐month history of eczema localized to the trunk and limbs. After clinical and histological evaluation, adult‐onset AD was diagnosed. Ten years before, chronic kidney disease had been diagnosed and treated with hemodialysis. At the time of the visit, the patient was on a renal transplant waiting list. Dermatological examination revealed a severe widespread AD (Eczema Area and Severity Index [EASI]:30) with intense pruritus (Pruritus‐Numerical Rating Scale [P‐NRS]:10) and negative impact on sleep (sleep(S)‐NRS:8) and quality of life (Dermatology Life Quality Index [DLQI]:22). In January 2021, labeled dose of dupilumab was started. At 4‐week follow‐up visit, patient showed significant improvements of AD (EASI:10; P‐NRS:5; S‐NRS:4; DLQI:5), reaching almost complete remission of signs and symptoms after 20‐week therapy (EASI:5; P‐NRS:2; S‐NRS:0; DLQI:2). In June 2021, dupilumab was suddenly discontinued, because patient underwent cadaveric renal transplantation and started anti‐rejection immunosuppressive therapy with everolimus 3 mg/day and tacrolimus 3 mg/day. In September 2021, the patient showed a relapse of AD (EASI:17; P‐NRS:8; S‐NRS:6; DLQI:12). Therefore, based on the benefit–risk assessment and in coordination with the transplant team, we restarted the administration of dupilumab with a significant skin clearance already after 1 month (EASI:2; P‐NRS:0; S‐NRS:0; DLQI:2). Currently, transplant‐related outcomes are good, and the patient is in stable conditions.

The second case is that of an 18‐year‐old female affected with AD since the first months of life. At the age of 1 year, she had undergone liver transplant surgery for congenital biliary atresia and was currently treated with tacrolimus 3 mg/day and mycophenolate mofetil 3 g/day. At our first visit, she presented with diffuse itchy eczema (EASI:28; P‐NRS:8; S‐NRS:7; DLQI: 27). Therapy with dupilumab at labeled dosage was started. After 16 weeks, the patient's considerably improved (EASI:3; PNRS:1; S‐NRS:0; DLQI: 5). The patient is treated since 3 years with dupilumab, without any apparent influence of dupilumab on liver function, while the skin improvement is constant over time.

Our data seem to confirm that dupilumab treatment can be practiced in transplant patients without apparent interference on efficacy and safety of the drug. The first patient underwent transplantation during dupilumab therapy, with a short suspension only in the phase immediately following the surgery. Dupilumab maintained its effectiveness even after transplantation. The efficacy and safety of dupilumab is also confirmed in the second case with a transplant during the first months of life. Our data also suggest that does not seem to be any correlation with the type of transplant, the time of surgery, and other immunosuppressants. Dupilumab can modulate the increased T‐helper (Th)2 response described in post‐transplantation.^8^ Indeed, post‐transplant immunosuppressive drugs (like systemic tacrolimus) was associated with a shift of the immune system towards the Th2 axis response through inhibition of Th1 cytokines, resulting in post‐transplant allergy, autoimmunity, and immune related disorder (PTAA).^8^ PTAA include urticaria/angioedema, eosinophilia, asthma, food allergies, and AD.^8^

Available reports and our experience seem to be reassuring on dupilumab use in transplant patients, also in those undergoing transplant. Obviously, a longer follow‐up period, as well as further and larger studies are needed to rule out any potential risk of organ rejection or alterations in organ function during dupilumab therapy and to evaluate long‐term efficacy and safety.

## CONFLICT OF INTEREST

Gabriella Fabbrocini acted as a speaker or consultant for Abbvie, Amgen, Eli Lilly, Janssen, Leo‐Pharma, Almyrall, Novartis, and UCB. None of the contributing authors has any conflict of interest, including specific financial interests of relationships and affiliation relevant to the subject matter or discussed materials in the manuscript.

## Data Availability

Data sharing is not applicable to this article as no new data were created or analyzed in this study.
